# Academic Burnout in University Students with Specific Learning Disorders: The Mediating Role of Anxiety in the Relationship Between Burnout and Depression

**DOI:** 10.3390/jcm14186400

**Published:** 2025-09-10

**Authors:** Michela Camia, Matteo Reho, Elisabetta Ferrari, Claudia Daria Boni, Valentina Ferretti, Giacomo Guaraldi, Elisabetta Genovese, Giorgia Varallo, Erika Benassi, Alessia Scarano, Valentina Baldini, Angela Ciaramidaro, Maristella Scorza

**Affiliations:** 1 Department of Biomedical, Metabolic and Neural Sciences, University of Modena and Reggio Emilia, Viale A. Allegri 9, 42121 Reggio Emilia, Italy; matteo.reho@unimore.it (M.R.); elisabetta.ferrari@unimore.it (E.F.); g.varallo@unimore.it (G.V.); ascarano@unimore.it (A.S.); valentina.baldini7@unibo.it (V.B.); angela.ciaramidaro@unimore.it (A.C.); maristella.scorza@unimore.it (M.S.); 2 Private Practice Neuropsychologist and Psychotherapist, via Monti 1, 42122 Reggio Emilia, Italy; claudiadaria.boni@gmail.com; 3 Private Practice Psychotherapist, via J.F. Kennedy 17, 42124 Reggio Emilia, Italy; valentina.ferretti7@unimore.it; 4 Specific Learning and Disabilities Service, University of Modena and Reggio Emilia, Strada Vignolese 671/a, 41125 Modena, Italy; giacomo.guaraldi@unimore.it; 5 Audiology Program, Department of Medical and Surgical Sciences for Children and Adults, University of Modena and Reggio Emilia, via del Pozzo, 71, 41124 Modena, Italy; elisabetta.genovese@unimore.it; 6 Department of Education and Humanities, University of Modena and Reggio Emilia, Viale Timavo 93, 42121 Reggio Emilia, Italy; erika.benassi@unimore.it; 7 Department of Biomedical and Neuromotor Sciences, University of Bologna, Viale C. Pepoli 5, 40123 Bologna, Italy

**Keywords:** academic burnout, Specific Learning Disorders, anxiety, depression

## Abstract

**Background**: The number of students with Specific Learning Disorders (SLDs) in universities has recently increased. Thus, it is important to analyze their difficulties throughout their academic studies and propose adequate interventions to prevent emotional problems and dropout. Previous research has reported higher levels of internalizing problems (anxiety and depression) in students with SLDs compared to those with typical development. Surprisingly, academic burnout among students with SLDs remains a largely overlooked and under-researched issue. The present work is one of the first studies that seeks to address this critical gap by examining the levels of academic burnout, and exploring its relationship with depression and anxiety in university students both with and without SLDs. **Methods**: The sample included 120 university students (M = 42, F = 78; mean age = 21.16, SD = 2.26). Of these, 60 students had SLDs and 60 had typical development (TD). Students were asked to complete three questionnaires assessing burnout (BAT-C), depression (BDI-II), and anxiety (STAI-Y). **Results**: The comparison between groups revealed that students with SLDs reported significantly higher levels of total burnout (mean difference = −3.98, t[118] = −2.59, *p* = 0.011, d = 0.47) and trait anxiety (mean difference = −2.87, t[118] = −2.73, *p* = 0.007, d = 0.50), with a moderate effect size for both differences. They also exhibited greater cognitive impairment related to burnout (U = 2333.50, *p* = 0.006, r = 0.25). No group differences were found in depression. Path analyses showed that while trait anxiety mediated the burnout–depression link in both groups, state anxiety was a significant mediator only for students with SLDs (β = 0.22, *p* = 0.025). **Conclusions**: The findings provide new evidence of the importance of monitoring academic burnout and anxiety in students with SLDs. The results show that anxiety plays a crucial mediating role between burnout and depression in students with SLDs, reinforcing the need for specific psychological support programs in universities.

## 1. Introduction

Specific Learning Disorders (SLDs) are among the most prevalent neurodevelopmental disorders (5 to 15%), involving difficulties in reading, spelling, writing, and mathematics in spite of adequate intelligence, intact sensory abilities, and appropriate instruction [1]. SLDs are lifelong conditions that persist in adulthood. Although students may show improvements as they age, some difficulties continue in professional and academic settings [2], such as problems in planning, task monitoring, and organization [3,4] and peculiar cognitive profiles [5,6]. These difficulties trigger lower quality of life, difficulties in academic self-efficacy, and lower psychological well-being [7,8,9,10,11]. Students with SLDs have been shown to experience higher levels of internalizing symptoms than their peers without SLDs. In particular, researchers found that dyslexic students reported higher levels of anxiety and depression, and they often felt more academically inadequate than their peers [12,13,14,15,16,17]. Moreover, during exams that tested reading abilities, university students with dyslexia showed higher levels of anxiety, both trait and state [13]. Surprisingly, while anxiety and depression have been extensively investigated in students with SLDs, the literature lacks studies on burnout in this population. To the best of our knowledge, only one study investigated burnout in students with SLDs [18]. The authors found higher levels of stress and burnout in students with SLDs compared to typically developing students.

Burnout consists of a long-term response to chronic stress, associated with depressive symptoms, a sense of helplessness, and detachment [19]. Burnout has more recently been applied to students, as school or university is considered the place where students “work” [20]. The concept of burnout in students derives from the work-related burnout construct and has been defined as a stress response when students are unable to cope with academic demands [21]. Academic burnout is a complex phenomenon influenced by multiple factors (such as academic stress, academic anxiety, and a lack of academic self-efficacy) that interact in contributing to its development [22].

School and academic burnout is characterized by exhaustion due to learning demands, a cynical and detached attitude, and a pessimistic sense of inadequacy, such as a low sense of efficacy [21,23,24]. As a result, students experiencing burnout show disinterest in school and academic material, increased absences from school, and academic failure [25].

Some students experience temporary stress during their compulsory schooling, but burnout is more likely to occur during university. In fact, university is a crucial stage of personal development associated with a higher pressure to acquire knowledge and new skills [26]. Most studies on burnout have investigated this construct in medical students or healthcare professionals, showing a high prevalence of burnout among medical students [27,28,29,30]. In a recent study that included 506 students from the first years of different undergraduate programs, the prevalence of burnout was 7.3%. The data were replicated and expanded by Liu [26], who investigated academic burnout in 22,983 Chinese students. Among the total sample, 40.01% of students reported no burnout, 55.16% had mild burnout, 3.55% had serious burnout, and 1.28% had very serious burnout.

School burnout is strongly linked to depression and anxiety, particularly among adolescents and university students. Research has shown that burnout serves as a risk factor for later depressive symptoms, as students struggling with academic demands may lack the resources to cope, making them more vulnerable to depression [30,31]. In university students, high stress, emotional fatigue, and low coping skills contribute to a loss of motivation and increased depressive symptoms [26]. Wang et al. [32] found that cynicism, particularly a lack of interest in studies, plays a central role in burnout and its connection to depression.

The association between burnout and anxiety seems to be strong, particularly during exams, as feelings of frustration and helplessness can exacerbate anxious symptoms [33]. Interestingly, Sun et al. [34] further demonstrated that anxiety could mediate the association between burnout and depression, suggesting a strong connection between academic stress and mental well-being.

Given the importance of burnout in psychological well-being and the lack of studies on burnout in university students with SLDs, the present study aims to investigate burnout in these specific participants compared to typically developing peers (TD). Second, we explored depression and anxiety in university students with and without SLDs, focusing on their relationship with burnout. The role of anxiety in the relationship between burnout and depression was explored with path analyses. In both groups, we hypothesized a direct and indirect effect, through anxiety, from burnout to depression. However, we expected stronger relationships in SLD students.

## 2. Materials and Methods

### 2.1. Participants and Procedure

One hundred and twenty university students (M = 42, F = 78) between the ages 18 and 30, with a mean age of 21.16 (SD = 2.26), took part in the study. Of these, 60 had a diagnosis of SLDs (mean age = 20.92, SD = 2.40; 35% males) and 60 were TD students (mean age = 21.40, SD = 2.10; 35% males).

The minimum sample size required for the path analysis was calculated following Cohen’s guidelines [35,36]. For a model with three paths predicting the dependent variable, and assuming a medium effect size (R^2^ = 0.25), 80% statistical power, and a 5% alpha error rate, a minimum of 59 participants was needed. This requirement was applied to the overall sample, as well as to the SLD and TD groups separately.

Students with SLDs were recruited from the Specific Learning and Disabilities Service of the University. All SLD students had a formal diagnosis made by public or private health services. The diagnosis of SLD was based on the criteria included in the ICD-10 coding system [37], and it conformed to the norms reported in the National Italian Consensus Conference on SLDs published by the Italian Ministry of Health [38]. The control group included undergraduate and graduate students from different university courses. TD students were invited to participate via notices posted at the university and via the university mailing list. To exclude any possible difficulty due to specific medical conditions, we set the presence of major neurological or psychiatric disorders and visual or hearing impairments as exclusion criteria. These data were obtained through an online questionnaire proposed on a Google Form. Additionally, all participants were native Italian speakers. All the students who agreed to participate provided an informed consent for privacy protection disclaimer before submitting the questionnaires. The anonymity was guaranteed by using Self-Generated Identification Codes. The study met the ethical guidelines for human subject protection, including adherence to the legal requirements of the country (Declaration of Helsinki), and it received formal approval by the local research Ethical Committee of the University of Modena and Reggio Emilia (protocol no. 2024-UNMRCLE-0178431, 17 May 2024).

### 2.2. Self-Report Measures

An ad hoc questionnaire was administered to collect the socio-demographic characteristics of the participants (gender, age, presence/absence of a diagnosis of SLDs).

The Beck Depression Inventory (BDI) [39,40] is a self-report measure assessing symptoms of depression. It includes 21 items that are rated on a 4-point Likert scale (e.g., “I do not feel sad” to “I am so sad or unhappy that I can’t stand it”). Higher scores indicate higher levels of depression. The BDI has been shown to possess good psychometric properties across clinical and community samples [40].

The STAI-Y [41,42] is a self-report questionnaire that includes two separate 20-item scales, measuring state anxiety (STAI-Y 1) and trait anxiety (STAI-Y 2). STAI-Y 1 reflects a temporary response to an event perceived as stressful, characterized by feelings of tension, nervousness, worry, and apprehension. In contrast, STAI-Y 2 represents a more stable tendency to view stressful situations as threatening or dangerous. The STAI-Y 1 scale assesses how the individual feels “right now, at this moment”, while the STAI-Y 2 scale evaluates how the individual feels “generally”. Both scales use a 4-point Likert scale (1–4), where a score of 4 indicates a higher level of anxiety for ten items on the STAI-Y 1 scale (3, 4, 6, 7, 9, 12, 13, 14, 17, 18) and eleven items on the STAI-Y 2 scale (2, 4, 5, 8, 9, 11, 12, 15, 17, 18, 20). For the remaining items, the score is reversed. The total score for each scale ranges from 20 to 80, with higher scores indicating more severe anxiety.

The Burnout Assessment Tool–Core Symptoms short version [43,44] was used to assess burnout. The BAT-C is one of the most widely used and validated tools in the Italian context for assessing burnout in students. It is composed of 12 items, rated on a five-point Likert scale (from 1 = Never to 5 = Always), which refer to four dimensions of burnout: exhaustion (items 1 to 3; e.g., “I feel mentally exhausted”), mental distance (items 4 to 6; e.g., “I struggle to find enthusiasm for studying”), cognitive impairment (items 7 to 9; e.g., “I have trouble staying focused”), and emotional impairment (items 10 to 12; e.g., “I feel unable to control my emotions”).

### 2.3. Data Analysis

To examine differences between the SLD and TD groups on the BAT-C, STAI-Y1, and STAI-Y2 scores, we used independent samples *t*-tests. However, the distribution of the BAT-C dimensions and the BDI violated the assumption of normality. Consequently, for these variables, the non-parametric alternative, the Mann–Whitney U test, was employed.

The core analysis tested a path model with BAT-C as the independent variable, BDI as the dependent variable, and STAI-Y1 and STAI-Y2 as mediators. This model was tested on the overall sample and separately for the SLD and TD groups. Given the specific characteristics of our data and model, we selected Partial Least Squares Structural Equation Modeling (PLS-SEM) [36] over traditional covariance-based SEM for three key reasons. First, the dependent variable (BDI) was not normally distributed, and PLS-SEM is a non-parametric technique that does not rely on distributional assumptions, making it robust in such scenarios [45]. Second, our sample size, particularly when divided into subgroups, was limited relative to the complexity of our model, which comprised 4 latent constructs and 53 indicators. PLS-SEM is well-suited for achieving statistical power with smaller samples and complex models [36]. Finally, PLS-SEM is ideal for models aimed at prediction and theory development, which aligns with the exploratory nature of our path analysis.

To compare the path coefficients of the tested model between the SLD and TD groups, a multi-group analysis (MGA) was performed [46,47]. Prior to the MGA, we established measurement invariance using the three-step procedure for composite models (MICOM) [46,48] to ensure that any observed differences in the model paths were due to structural differences and not measurement discrepancies.

All model estimates were validated using a robust bootstrapping procedure with 5000 bootstrap samples and the bias-corrected and accelerated (BCa) method [36,49].

The t-tests and Mann–Whitney U tests were performed using SPSS software version 26.0 [50]. The PLS-SEM analysis, including path analysis and MGA, was conducted using SmartPLS 4 software [51].

## 3. Results

### 3.1. Groups Comparisons

A Mann–Whitney U test performed to evaluate possible differences in the age of the participants did not yield statistically significant results (U = 1485, *p* = 0.094). As shown in Table 1, the groups comparison revealed that the participants in the SLDs group reported significantly higher scores than the participants in the TD group on the BAT-C scale (mean difference = −3.98, t[118] = −2.59, *p* = 0.011, d = −0.47). The cognitive impairment dimension of the BAT-C also showed significant differences, with higher levels in the SLDs group (mean rank difference = −17.42, U = 2333.50, *p* = 0.006, r = 0.29). No significant differences emerged for the BAT-C exhaustion (U = 2164, *p* = 0.055), mental distance (U = 2111.50, *p* = 0.099), or emotional impairment (U = 1864, *p* = 0.735) dimensions. A difference emerged between the groups in the STAI-Y2 scores, with the SLDs group reporting higher levels of trait anxiety (mean difference = −2.87, t[118] = −2.73, *p* = 0.007, d = −0.50). No differences emerged in the STAI-Y1 scores for state anxiety (t[118] = −0.14, *p* = 0.887). No significant difference emerged between the groups in the BDI scores (U = 1975, *p* = 0.357).

### 3.2. Measurement Model

The measurement model was assessed by analyzing the reliability of the indicators, the internal consistency, and the convergent and discriminant validity for all the samples considered (overall, SLDs, TD). The reliability of the indicators was evaluated by inspecting their loadings. Indicators 4 and 6 of the BAT-C, indicators 9, 10, 12, 18, and 21 of the BDI, indicator 19 of the STAI-Y1, and indicators 14, 19, and 20 of the STAI-Y2 were excluded because they did not reach the minimum threshold (λ ≥ 0.40), according to the recommendations of Hair et al. [36]. All indicators with loadings equal to or greater than 0.40 were retained (see Table S1 in the Supplementary Materials), as their removal would not have resulted in an improvement in internal consistency [52].

The reliability of the internal consistency was measured using Cronbach’s alpha coefficient (α) and the composite reliability index (rho-c). As reported in Table S2 in the Supplementary Materials, the values of α and rho-c exceeded the minimum threshold of 0.70 without exceeding the maximum limit of 0.95, indicating an adequate level of internal consistency [52]. Convergent validity was examined by calculating the average variance extracted (AVE), considering values equal to or greater than 0.50 to be acceptable. As shown in Table 2, in the present study, only the STAI-Y1 exceeded this threshold. However, according to Fornell and Larcker [53], if the AVE is less than 0.50 but the rho-c exceeds 0.60, it is still possible to confirm the convergent validity of the construct (see Table S2).

Finally, the discriminant validity was evaluated through the heterotrait–monotrait ratio (HTMT). As shown in Table S3 in the Supplementary Materials, all HTMT values were below the threshold of 0.85 for distinct constructs and 0.90 for conceptually similar constructs, according to the criteria established by Henseler et al. [54]. The only exceptions were the comparisons between STAI-Y2 and BDI in the SLDs group, and between STAI-Y1 and STAI-Y2 in the TD group, whose HTMT values slightly exceeded the respective thresholds.

### 3.3. Path Model

To evaluate the path model, the Variance Inflation Factor (VIF) was examined to identify collinearity issues, together with the path coefficients and the determination coefficient R2 for each sample. As shown in Table S4 in the Supplementary Materials, all VIF values were below the threshold of 5 suggested by Hair et al. [52], indicating the absence of collinearity between the variables in the three samples considered.

**Figure 1:**
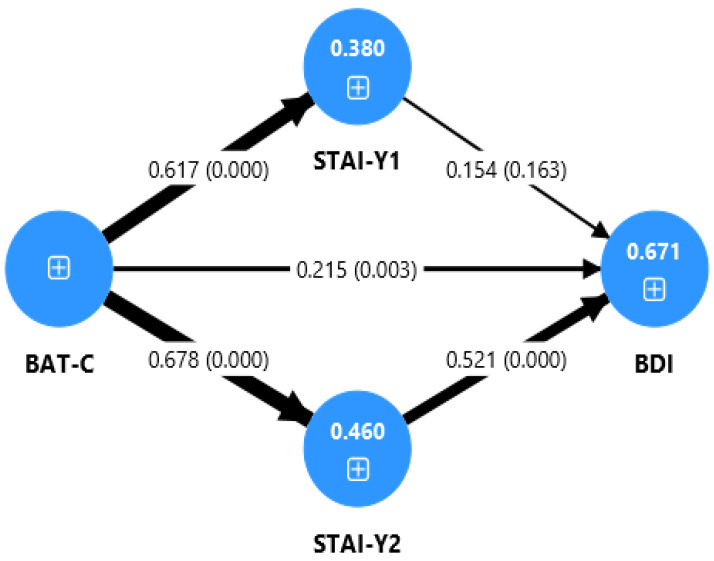
Path model for the overall sample. BAT-C: Burnout Assessment Tool—Core Symptoms; BDI: Beck Depression Inventory; STAI-Y1: State-Trait Anxiety Inventory—Form Y1 (State); STAI-Y2: State-Trait Anxiety Inventory—Form Y2 (Trait); SLDs: Specific Learning Disabilities; TD: Typical Development. The thickness of the arrows corresponds to the strength of the relationships between the variables. Values represent path coefficients (β) and *p*-values (in parentheses).

In the overall sample (Figure 1), burnout had a significant direct effect on depression (β = 0.21, *p* = 0.003). The indirect effect through trait anxiety was significant and strong (β = 0.35, *p* < 0.001), accounting for a substantial portion of the total effect. The indirect effect through state anxiety was not significant.

**Figure 2:**
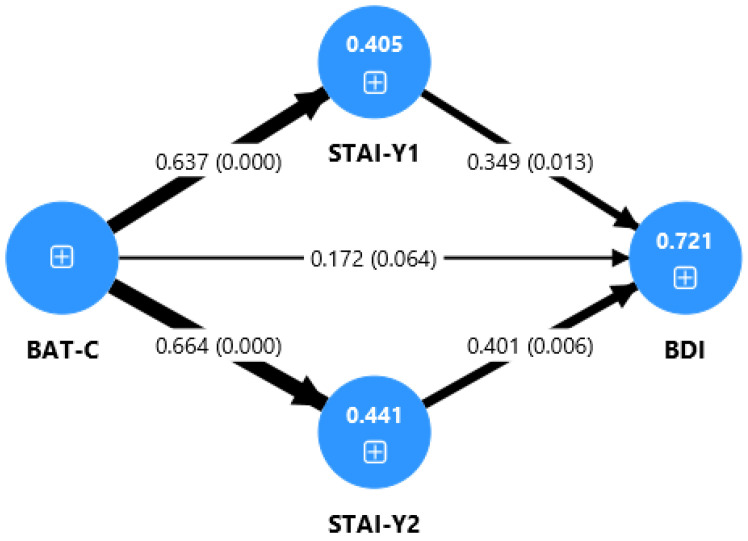
Path model for the SLDs sample. BAT-C: Burnout Assessment Tool—Core Symptoms; BDI: Beck Depression Inventory; STAI-Y1: State-Trait Anxiety Inventory—Form Y1 (State); STAI-Y2: State-Trait Anxiety Inventory—Form Y2 (Trait); SLDs: Specific Learning Disabilities; TD: Typical Development. The thickness of the arrows corresponds to the strength of the relationships between the variables. Values represent path coefficients (β) and *p*-values (in parentheses).

In the SLDs group (Figure 2), the direct effect of burnout on depression was not significant (β = 0.17, *p* = 0.064). Instead, the relationship was fully explained by the indirect effects through both state anxiety (β = 0.22, *p* = 0.025) and trait anxiety (β = 0.27, *p* = 0.012).

**Figure 3:**
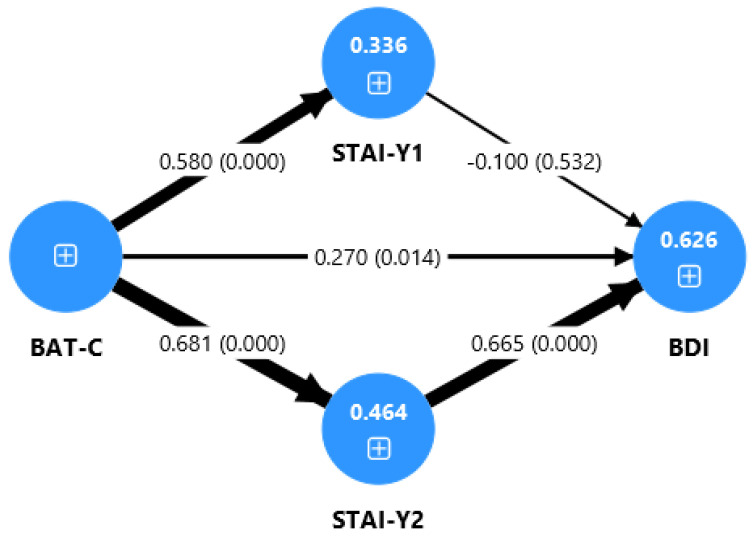
Path model for the STD sample. BAT-C: Burnout Assessment Tool—Core Symptoms; BDI: Beck Depression Inventory; STAI-Y1: State-Trait Anxiety Inventory—Form Y1 (State); STAI-Y2: State-Trait Anxiety Inventory—Form Y2 (Trait); SLDs: Specific Learning Disabilities; TD: Typical Development. The thickness of the arrows corresponds to the strength of the relationships between the variables. Values represent path coefficients (β) and *p*-values (in parentheses).

Finally, in the TD group (Figure 3), burnout had a significant direct effect on depression (β = 0.27, *p* = 0.014). The indirect effect was significant only through trait anxiety (β = 0.45, *p* < 0.001), which was the strongest effect observed across all groups.

The model explained a large portion of the variance in depression scores, particularly in the SLDs group (R^2^ = 0.72) compared to the TD group (R^2^ = 0.63). The path coefficients and bootstrap confidence intervals are detailed in Table 2, and the indirect effects are summarized in Table 3.

Figure 1, Figure 2 and Figure 3 present the tested path model for the three samples considered, displaying the standardized path coefficients (β) and the coefficients of determination (R^2^) for the overall sample, the SLDs group, and the TD group. The thickness of the arrows corresponds to the strength of the relationships between the variables.

As reported in Table 3, the mediation analysis (Figure 1) showed that, in the SLDs group, the indirect effect of BAT-C on BDI, mediated by STAI-Y1, was significant (β = 0.22, t = 2.25, *p* = 0.025). Furthermore, for all samples, a significant indirect effect of BAT-C on BDI was found through the mediation of STAI-Y2 (overall: β = 0.35, t = 4.20, *p* < 0.001; SLDs: β = 0.27, t = 2.52, *p* = 0.012; TD: β = 0.45, t = 3.92, *p* < 0.001), with a higher effect in the TD group.

Finally, the R^2^ values indicate that the BAT-C explained a significant portion of the variance in the BDI, STAI-Y1, and STAI-Y2 scores in the different samples analyzed. Specifically, in the overall sample, the BAT-C explained 67% of the variance in the BDI, 38% of the STAI-Y1, and 46% of the STAI-Y2. Considering the groups separately, the effect of BAT-C on BDI was higher in the SLDs group (R^2^ = 0.72) than in the TD group (R^2^ = 0.63). For the STAI-Y1, the explained variance was 41% in the SLDs group and 34% in the TD group. Finally, for the STAI-Y2, the R^2^ values were almost the same in both groups (SLDs: R^2^ = 0.44; TD: R^2^ 0.46).

### 3.4. Multigroup Analysis

Before conducting the MGA to compare the differences in path coefficients between the SLDs and TD groups, the MICOM three-step procedure was applied to verify measurement invariance and exclude potential bias between groups [48].

The first step in the MICOM procedure concerns configural invariance, which ensures that indicators, data processing, and algorithms are identical for each sample. In SmartPLS 4, this setting is predefined for all samples. Second, through the permutation algorithm, compositional invariance was evaluated, which is considered satisfied when the original correlation between the constructs is greater than or equal to the value of the 5th percentile of the permuted distribution [Matthews]. As reported in Table S5 in the Supplementary Materials, all the original correlation values exceeded or were equal to the 5% threshold, thus satisfying this criterion.

The third step involves calculating the composite equality, which is considered fully satisfied (full invariance) if both the original mean difference and the original variance difference fall within the 95% confidence interval. If only one of these two criteria is met, it is referred to as partial invariance, while the absence of both indicates non-invariance. As shown in Table S6 in the Supplementary Materials, in the present study, only the STAI-Y1 met the criterion of full invariance, while no invariance was found for the other variables.

Based on these results, MGA was performed to compare the STAI-Y1 -> BDI path coefficient in the SLDs and TD groups. The analysis showed a significant difference between the two groups (difference = 0.45, *p* = 0.048, 95% CI: 2.5% = −0.45, 97.5% = 0.44), indicating a greater effect in the SLDs group (β = 0.35) compared to the TD group (β = −0.10). This result indicates that the SLDs group moderates the observed relation.

## 4. Discussion

The main goal of the present study was to assess burnout in a group of university students. We investigated, for the first time, the levels of burnout in students with SLDs compared to TD students. Our findings indicate a higher level of burnout in university students with SLDs than in their TD peers (see Table 1). Previous studies have found that, among typically developing university students, burnout is linked to lower academic performance and often leads to reduced concentration and frequent distracting thoughts (e.g., [55]). The difficulties that students with SLDs face during their studies seem to lead to greater challenges in managing academic stress and fatigue, consequently resulting in burnout. Moreover, among the four scales of burnout, we found a significant difference in the scale evaluating cognitive impairments related to difficulties in attention, concentration, and memory (see Table 1). This difference concerns an area of functioning characterized by vulnerabilities in students with SLDs, even in conditions of psychological well-being, namely executive functions. Students with SLDs indeed show difficulties in working memory, speed processing, and executive functions [3,4,56]. Our results suggest that the presence of SLDs seems to increase these conditions. To better understand these difficulties, we also analyzed anxiety and depression. First, we found no differences in symptoms of depression when comparing SLD students with a matched control group. Our data do not replicate the overall results presented in previous studies that reported an association between dyslexia and depression [16]. However, although the difference was not statistically significant, the results showed severe depressive symptoms only in the SLDs group. Our findings may be explained by considering that students with SLDs were able to use compensatory tools and had access to dedicated support services for learning disorders. These resources could be considered protective factors and could help explain the results observed in the SLD group.

Regarding anxiety, the current study indicated a difference between SLD and TD students in trait anxiety, whereas no differences were found in state anxiety (see Table 1). General anxiety symptoms have been frequently reported by university students during exams [57], with increasing levels during the semester [58]. Exam anxiety is a significant issue for university students, often linked to a decrease in performance and psychological challenges, even among those with typical development [59]. On the contrary, students with SLDs showed higher levels of trait anxiety; thus they tended to evaluate life situations, not just academic ones, as threatening. Our data are consistent with previous studies that reported a consistent contribution of reading difficulties to anxiety in students from the first grade of university [11,17,60,61]. In fact, learning challenges lead students with SLDs to perceive lower self-esteem [62,63] and self-efficacy [64], increasing the risk of anxiety [65]. Moreover, Kajastus [66] argued that the difficulties experienced at school may affect other areas of daily life and represent a risk factor for the stabilization of anxiety symptoms.

In line with the previous literature, our findings highlight the impact of anxiety on the psychological well-being of university students with SLDs. The presence of SLDs is associated not only with test-related anxiety but also with a more generalized state of anxiety, which can affect various aspects of emotional life and daily functioning [67]. It is therefore important to implement annual screenings to assess anxiety levels in all students, particularly those with SLDs, and to establish psychological support programs to help students cope more effectively with studying and learning.

Moreover, we found a relationship between burnout and anxiety both in SLDs and in TD students (see Table 2, Figure 1 and Figure 2). According to our results, it seems that students who are more prone to experiencing burnout are also more likely to develop higher levels of anxiety (trait and state anxiety). The burnout–anxiety relationship has been previously reported in studies on adults that found a strong link between the two variables. In a recent review, Koutsimani and colleagues [68] found that, in adults, job and social demands exacerbate burnout and anxiety symptoms, which are significantly correlated with one another. Our results are consistent with the previous literature, indicating that also in university students, burnout and anxiety are interconnected and likely develop concurrently.

The present study also examined the association between academic burnout and depression. In the total group (SLDs and control group) and in the control group, we found a direct effect of burnout on depression (see Table 2 and Figure 1 and Figure 3). The link between academic burnout and depression has been previously reported in studies on adolescents and university students [23,31]. School or academic burnout could have the effect of depressive symptoms, as students’ exhaustion, cynicism, and feelings of inadequacy contribute to an increase in depressive symptoms. In fact, if students experience emotional exhaustion from dealing with the challenges of school life, they are more likely to feel fatigued and to be more irritable and frustrated [21]. However, in the SLDs group, we found only an indirect effect of burnout on depression, through the mediation of anxiety (see Table 2 and Figure 2).

Our hypothesis is that university students with SLDs have a greater ability to cope with negative emotions related to the academic context and are more tolerant of disappointments, making them more protected from the consequences of burnout. In fact, our data showed that SLD students develop depressive symptoms only in the presence of anxiety that makes it more difficult to face the challenges of academic life, increases the negative emotions of burnout, and consequently leads the student to experience depressive symptoms. Interestingly, Fiorilli [23] found a joint effect of burnout, depression, and engagement on two students’ achievement, i.e., students’ average grades and levels of absenteeism. Engagement includes three dimensions, vigor, dedication, and absorption [23], and it has been considered a risk factor for both depression and negative emotions toward learning. According to previous studies, university students with SLDs had high levels of determination, worked with greater effort as a consequence of their difficulties [69], showed good awareness of their challenges, and had developed effective coping strategies [70]. Thus, in facing everyday challenges due to their difficulties, they are more prone to developing good engagement. Moreover, according to Aro [71], having a specific learning difficulty might contribute to greater resilience toward negative events. The data from the present study seem to support these last findings, namely that students with SLDs, as a result of their educational history and the challenges they have consistently faced, may develop more effective coping strategies for dealing with academic demands, failures, and difficulties. Nevertheless, the presence of anxiety appears to compromise psychological functioning, thereby reducing the capacity to buffer against the onset of depressive symptoms.

Regarding the role of anxiety in the relationship between burnout and depression, through the path model tested, we found that in the total group and the clinical group, the only mediator is trait anxiety. By contrast, in the SLDs group, both state and trait have an effect on the link between burnout and depression (see Table 2 and Figure 2). The strong relationship between trait anxiety and depression has been described in previous studies on typically developing adults [72]. This study is therefore consistent with the data from other research and supports the role of anxiety in the onset of depressive symptoms. Having high levels of anxiety is thus a risk factor that contributes to difficulties in managing situations of frustration, fatigue, and stress, leading to depressive symptoms. By contrast, state anxiety seems to be a risk factor only for students with SLDs. According to our data, anxiety related to specific moments in academic life, such as exams, leads to depression only in the presence of other difficulties that make the student more vulnerable. This last finding is also supported by the multigroup analysis performed, which reveals a significant difference between Sthe LD and TD groups only for state anxiety. Specifically, in the SLD students only, state anxiety has an effect on the relationship between burnout and depression, while the role of trait anxiety is strong in both the SLDs and control groups.

The strength of the relationships observed in our model aligns with different studies while also highlighting the specific vulnerability of the SLDs group. For example, the direct effect of burnout on depression in the overall sample is identical to the path coefficient reported by Sun et al. [34] in a sample of Chinese university students (β = 0.21). However, it is notably lower than the strong total effect (β = 0.51) found by Fiorilli et al. [23] in a study on high school students. This discrepancy may be attributed to developmental differences or the use of different analytical techniques (PLS-SEM vs. CB-SEM). Crucially, the mediating effect of state anxiety in the SLDs group (β = 0.22), a core finding of our study, demonstrates a meaningful effect size. While Sun et al. [34] found a much larger direct effect of anxiety on depression (β = 0.771), their model did not examine the specific mediating role of state anxiety in a clinical group like ours. Therefore, our results provide a more nuanced understanding, suggesting that for students with SLDs, the pathway from burnout to depression is not direct, but is significantly channeled through anxious states, with an effect size that underscores its clinical relevance.

Overall, our data on burnout and on the role that anxiety may play in the onset of subsequent depressive symptoms show, for the first time, the necessity of assessing the emotional functioning profile of students, particularly in the presence of neurodevelopmental disorders such as SLDs. This study highlights the importance of a comprehensive evaluation of psychological well-being and of the potential impact that academic difficulties could have on students’ lives. Moreover, the findings provide initial evidence that can inform the development of psychological support programs and interventions for students, starting from their first year of university.

### Limitations

The current study had some limitations that should be mentioned and taken into account in future research. First, the data were collected using online self-reported questionnaires, which may be susceptible to social desirability bias. Second, protective factors should be investigated to better understand the pathways leading to burnout. In particular, higher levels of resilience and self-efficacy could prevent burnout [73]. Finally, a broader sample that includes students from other universities would have been valuable, as it would have allowed for better generalization of our findings to the wider population of students in Italy.

## 5. Conclusions

The current study is the first to investigate burnout in university students with SLDs, showing higher levels of burnout compared to TD students. Moreover, the data reveal a link between burnout and depression through the mediation of anxiety. Thus, higher levels of anxiety represent a significant risk factor for later depression in students. The results offer a more comprehensive perspective for research on university students’ mental health. This study suggests the importance of implementing an effective program to support students with SLDs in reducing burnout. Furthermore, given the link between burnout and depression in both SLD and TD students, this study provides some recommendations to psychologists suggesting that all students undergo an annual burnout and anxiety screening to prevent the development of later depressive symptoms. Moreover, this study highlights the importance of university professors considering the potential impact of SLDs on students’ learning and psychological well-being, encouraging the adoption of teaching strategies that can support students with SLDs in effectively managing their studies and exams.

## Figures and Tables

**Table 1:** Group comparisons of the scores for BAT-C, STAI-Y1, and STAI-Y2.

Variables	Group	Mean/ Mean Rank ^a^	Std. Deviation	Mean Difference/ Mean Rank Difference ^a^	T/U ^a^	*p*
BAT-C	TD	30.5	7.28	−3.98	−2.59	**0.011**
	SLDs	34.48	9.43			
BAT-C: EX ^a^	TD	54.43		−12.14	2164	0.055
	SLDs	66.57				
BAT-C: MD ^a^	TD	55.31		−10.38	2111.5	0.099
	SLDs	65.69				
BAT-C: CI ^a^	TD	51.79		−17.42	2333.5	**0.006**
	SLDs	69.21				
BAT-C: EI ^a^	TD	59.43		−2.14	1864	0.735
	SLDs	61.57				
BDI ^a^	TD	57.58		−5.84	1975	0.357
	SLDs	63.42				
STAI-Y1	TD	47.92	4.53	−0.13	−0.14	0.887
	SLDs	48.05	5.63			
STAI-Y2	TD	48.68	6.21	−2.87	−2.73	**0.007**
	SLDs	51.55	5.25			

Note. BAT-C: Burnout Assessment Tool—Core Symptoms; EX: exhaustion; MD: mental distance; CI: cognitive impairment; EI: emotional impairment; STAI-Y1: State-Trait Anxiety Inventory—Form Y1 (State); STAI-Y2: State-Trait Anxiety Inventory—Form Y2 (Trait); SLDs: Specific Learning Disabilities; TD: Typical Development. ^a^ Mann–Whitney U test was performed.

**Table 2:** Path coefficients of the relationships between the variables in the three samples.

							95% Confidence Interval	
Sample	Direct Effect	β	Mean	Std. Deviation	t	*p*	2.5%	97.5%
Overall	BAT-C -> BDI	0.21	0.21	0.07	3.02	**0.003**	0.08	0.36
	BAT-C -> STAI-Y1	0.62	0.63	0.06	10.55	**<0.001**	0.47	0.71
	BAT-C -> STAI-Y2	0.68	0.69	0.06	11.14	**<0.001**	0.52	0.77
	STAI-Y1 -> BDI	0.15	0.15	0.11	1.40	0.163	−0.07	0.36
	STAI-Y2 -> BDI	0.52	0.52	0.12	4.44	**<0.001**	0.29	0.74
SLDs	BAT-C -> BDI	0.17	0.17	0.09	1.85	0.064	−0.02	0.36
	BAT-C -> STAI-Y1	0.64	0.65	0.08	8.44	**<0.001**	0.42	0.75
	BAT-C -> STAI-Y2	0.66	0.68	0.09	7.70	**<0.001**	0.43	0.79
	STAI-Y1 -> BDI	0.35	0.35	0.14	2.50	**0.013**	0.07	0.61
	STAI-Y2 -> BDI	0.4	0.40	0.15	2.74	**0.006**	0.11	0.69
TD	BAT-C -> BDI	0.27	0.25	0.11	2.46	**0.014**	0.06	0.49
	BAT-C -> STAI-Y1	0.58	0.61	0.08	7.52	**<0.001**	0.39	0.69
	BAT-C -> STAI-Y2	0.68	0.70	0.06	11.3	**<0.001**	0.50	0.77
	STAI-Y1 -> BDI	−0.10	−0.09	0.16	0.63	0.532	−0.44	0.2
	STAI-Y2 -> BDI	0.66	0.68	0.15	4.30	**<0.001**	0.32	0.95

Note. BAT-C: Burnout Assessment Tool—Core Symptoms; BDI: Beck Depression Inventory; STAI-Y1: State-Trait Anxiety Inventory—Form Y1 (State); STAI-Y2: State-Trait Anxiety Inventory—Form Y2 (Trait); SLDs: Specific Learning Disabilities; TD: Typical Development.

**Table 3:** Indirect effect of BAT-C on BDI in the three samples.

							95% Confidence Interval	
Sample	Direct Effect	β	Mean	Std. Deviation	t	*p*	2.50%	97.50%
Overall	BAT-C -> STAI-Y2 -> BDI	0.35	0.36	0.08	4.20	**<0.001**	0.20	0.53
	BAT-C -> STAI-Y1 -> BDI	0.10	0.10	0.07	1.33	0.183	−0.05	0.24
SLDs	BAT-C -> STAI-Y2 -> BDI	0.27	0.27	0.11	2.52	**0.012**	0.07	0.49
	BAT-C -> STAI-Y1 -> BDI	0.22	0.23	0.10	2.25	**0.025**	0.03	0.42
TD	BAT-C -> STAI-Y2 -> BDI	0.45	0.48	0.12	3.92	**<0.001**	0.20	0.66
	BAT-C -> STAI-Y1 -> BDI	−0.06	−0.05	0.10	0.59	0.556	−0.26	0.13

Note. BAT-C: Burnout Assessment Tool—Core Symptoms; BDI: Beck Depression Inventory; STAI-Y1: State-Trait Anxiety Inventory—Form Y1 (State); STAI-Y2: State-Trait Anxiety Inventory—Form Y2 (Trait); SLDs: Specific Learning Disabilities; TD: Typical Development.

## Data Availability

The data presented in this study are available on request from the corresponding author. The data are not publicly available due to privacy reasons.

## Supplementary Materials

The following supporting information can be downloaded at https://www.mdpi.com/article/10.3390/jcm14186400/s1. Table S1. Factor loadings of the indicators on the respective constructs for the overall, SLDs and TD samples. Table S2. Values of the internal consistency indexes and convergent validity of the constructs in the overall sample, SLDs and TD groups. Table S3. HTMT values of the constructs in the overall sample, SLDs and TD groups. Table S4. Variance Inflation Factor (VIF) values for the mediation model in the different samples. Table S5. Compositional invariance of the analyzed constructs. Table S6. Composite equality of the analyzed constructs.
