# Supplementary material for: Academic Burnout in University Students with Specific Learning Disorders: The Mediating Role of Anxiety in the Relationship Between Burnout and Depression
Source: J Clin Med. 2025 Sep 10;14(18):6400. doi: 10.3390/jcm14186400 (PMC12470895; doi:10.3390/jcm14186400)
Supplement: Supplementary file 1 [file jcm-14-06400-s001.zip › jcm-3824576-supplementary.pdf]

Table S1. Factor loadings of the indicators on the respective constructs for the overall, SLDs and TD samples.

	Overall sample				SLD sample				TD sample			
Indicator	BAT-C	BDI	STAI-Y1	STAI-Y2	BAT-C	BDI	STAI-Y1	STAI-Y2	BAT-C	BDI	STAI-Y1	STAI-Y2
1	0.79	0.72	0.73	0.77	0.79	0.76	0.76	0.80	0.76	0.57	0.67	0.74
2	0.80	0.52	0.65	0.69	0.81	0.45	0.75	0.80	0.78	0.73	0.49	0.51
3	0.78	0.68	0.79	0.77	0.83	0.70	0.77	0.83	0.66	0.57	0.79	0.68
4	-	0.57	0.79	0.68	-	0.61	0.77	0.79	-	0.46	0.82	0.53
5	0.53	0.69	0.82	0.72	0.56	0.71	0.87	0.72	0.46	0.60	0.77	0.68
6	-	0.62	0.75	0.64	-	0.76	0.72	0.67	-	0.41	0.80	0.59
7	0.74	0.84	0.67	0.74	0.70	0.88	0.67	0.76	0.74	0.73	0.75	0.69
8	0.74	0.65	0.75	0.57	0.69	0.66	0.80	0.47	0.79	0.56	0.67	0.69
9	0.67	-	0.61	0.61	0.71	-	0.58	0.68	0.62	-	0.70	0.50
10	0.65	-	0.76	0.77	0.66	-	0.82	0.81	0.62	-	0.63	0.68
11	0.62	0.66	0.78	0.64	0.63	0.75	0.85	0.68	0.63	0.46	0.67	0.57
12	0.61	-	0.79	0.75	0.66	-	0.79	0.84	0.58	-	0.77	0.59
13		0.67	0.71	0.73		0.68	0.62	0.79		0.64	0.81	0.58
14		0.78	0.52	-		0.85	0.51	-		0.61	0.54	
15		0.67	0.80	0.77		0.72	0.79	0.85		0.45	0.79	0.59
16		0.53	0.73	0.77		0.50	0.73	0.81		0.57	0.72	0.74
17		0.59	0.81	0.59		0.67	0.77	0.62		0.43	0.86	0.49
18		-	0.70	0.57		-	0.66	0.62		-	0.76	0.46
19		0.70	-	-		0.70	-	-		0.69	-	-
20		0.71	0.78	-		0.77	0.85	-		0.55	0.66	-
21		-				-				-		

Note. The removed indicators are indicated by a dash. BAT-C: Burnout Assessment Tool – Core Symptoms; BDI: Beck Depression Inventory; STAI-Y1: State-Trait Anxiety Inventory – Form Y1 (State; STAI-Y2: State-Trait Anxiety Inventory – Form Y2 (Trait); SLDs: Specific Learning Disabilities; TD: Typical Development.

Table S2. Values of the internal consistency indexes and convergent validity of the constructs in the overall sample, SLDs and TD groups.

	Overall			SLDs			TD		
Construct	α	rho-c	AVE	α	rho-c	AVE	α	rho-c	AVE
BAT-C	0.88	0.90	0.49	0.89	0.91	0.50	0.86	0.89	0.45
BDI	0.91	0.93	0.44	0.93	0.94	0.50	0.86	0.88	0.33
STAI-Y1	0.95	0.95	0.54	0.95	0.95	0.56	0.95	0.95	0.53
STAI-Y2	0.93	0.94	0.48	0.95	0.95	0.55	0.89	0.91	0.38

Note. BAT-C: Burnout Assessment Tool – Core Symptoms; BDI: Beck Depression Inventory; STAI-Y1: State-Trait Anxiety Inventory – Form Y1 (State; STAI-Y2: State-Trait Anxiety Inventory – Form Y2 (Trait); SLDs: Specific Learning Disabilities; TD: Typical Development.

Table S3. HTMT values of the constructs in the overall sample, SLDs and TD groups.

	Overall	SLDs	TD
BDI <-> BAT-C	0.73	0.70	0.76
STAI-Y1 <-> BAT-C	0.66	0.67	0.62
STAI-Y1 <-> BDI	0.77	0.84	0.65
STAI-Y2 <-> BAT-C	0.73	0.70	0.75
STAI-Y2 <-> BDI	0.85	0.86	0.83
STAI-Y2 <-> STAI_Y1	0.89	0.89	0.91

Note. BAT-C: Burnout Assessment Tool – Core Symptoms; BDI: Beck Depression Inventory; STAI-Y1: State-Trait Anxiety Inventory – Form Y1 (State; STAI-Y2: State-Trait Anxiety Inventory – Form Y2 (Trait); SLDs: Specific Learning Disabilities; TD: Typical Development.

Table S4. Variance Inflation Factor (VIF) values for the mediation model in the different samples.

	Overall	SLDs	TD
BAT-C -> BDI	1.87	1.84	1.87
BAT-C -> STAI-Y1	1.00	1.00	1.00
BAT-C -> STAI-Y2	1.00	1.00	1.00
STAI-Y1 -> BDI	3.66	4.06	3.54
STAI-Y2 -> BDI	4.21	4.32	4.39

Note. BAT-C: Burnout Assessment Tool – Core Symptoms; BDI: Beck Depression Inventory; STAI-Y1: State-Trait Anxiety Inventory – Form Y1 (State; STAI-Y2: State-Trait Anxiety Inventory – Form Y2 (Trait); SLDs: Specific Learning Disabilities; TD: Typical Development.

Table S5. Compositional invariance of the analyzed constructs.

	Original correlation	Correlation permutation mean	5.0%	Permutation p value
BAT-C	0.99	0.99	0.98	.772
BDI	0.99	0.99	0.99	.080
STAI-Y1	0.99	0.99	0.99	.022
STAI-Y2	0.99	0.99	0.99	.292

Note. BAT-C: Burnout Assessment Tool – Core Symptoms; BDI: Beck Depression Inventory; STAI-Y1: State-Trait Anxiety Inventory – Form Y1 (State; STAI-Y2: State-Trait Anxiety Inventory – Form Y2 (Trait); SLDs: Specific Learning Disabilities; TD: Typical Development.

Table S6. Composite equality of the analyzed constructs.

		Original difference	Permutation mean difference	2.5%	97.5%	Permutation p value
Mean	BAT-C	0.44	-0.003	-0.35	0.34	.010
	BDI	0.35	0.003	-0.35	0.33	.046
	STAI-Y1	0.27	0.000	-0.37	0.34	.139
	STAI-Y2	0.42	-0.001	-0.36	0.36	.024
Variance	BAT-C	0.64	-0.005	-0.48	0.46	.004
	BDI	1.16	0.000	-0.81	0.73	.004
	STAI-Y1	0.36	-0.002	-0.43	0.44	.105
	STAI-Y2	0.81	-0.003	-0.47	0.45	< .001

Note. BAT-C: Burnout Assessment Tool – Core Symptoms; BDI: Beck Depression Inventory; STAI-Y1: State-Trait Anxiety Inventory – Form Y1 (State; STAI-Y2: State-Trait Anxiety Inventory – Form Y2 (Trait); SLDs: Specific Learning Disabilities; TD: Typical Development.
